# Gouty arthritis model: delving into disease pathways and uncovering possible therapeutic targets

**DOI:** 10.3389/fendo.2026.1752411

**Published:** 2026-04-13

**Authors:** Sanjin Zeng, Qiumei He, Rong Wang, Bingbing Chen, Dongxu Zhou, Na Zhou, Yong Liao, Shengyi Zhao, Zhaohu Xie, Ziyue Huang, Zhaofu Li, Jing Xie

**Affiliations:** 1 Yunnan University of Chinese Medicine, Kunming, China; 2 Kunming Municipal Hospital of Traditional Chinese Medicine, Kunming, China

**Keywords:** experimental models, gouty arthritis, *in vitro* model, *in vivo* model, innate immunity, programmed cell death

## Abstract

Gouty arthritis (GA) falls within the category of metabolic arthropathies. Its onset stems from abnormal uric acid metabolism, which subsequently leads to the deposition of monosodium urate (MSU) crystals and ultimately triggers a robust inflammatory response. Currently, the global prevalence rate of GA is on the rise, gradually increasing the societal disease burden it imposes. This review comprehensively examines the pathogenesis of GA. The content encompasses uric acid metabolic disorders, the innate immune activation process induced by MSU crystals, as well as various subsequently triggered programmed cell death (PCD) modalities, including pyroptosis, NETosis, apoptosis, necroptosis and ferroptosis. We then evaluate *in vivo* and *in vitro* experimental models according to the disease stage and pathogenic processes they best recapitulate. Exogenous MSU models are highly suitable for studying acute inflammatory flares; hyperuricemia models capture the metabolic basis of disease initiation; and composite models more closely reflect the chronic and multifactorial course of human gout. *In vitro* systems ranging from macrophage monocultures to co-culture and organoid platforms provide complementary tools for mechanistic studies and drug screening. However, current models still cannot fully reproduce the complexity of human gout, particularly with respect to metabolic initiation, tissue hierarchy, systemic context, and species-specific differences. We therefore propose a model-selection approach in which the choice of platform should be guided by the specific pathogenic process under investigation. Future model development should integrate innovative technologies to enhance the authenticity of pathological features, address the shortcomings of existing systems, and facilitate the clinical translation of GA research.

## Introduction

GA is an inflammatory joint disease that results from disordered uric acid metabolism. This condition subsequently induces the deposition of MSU crystals within joints and the surrounding tissues, which can further trigger inflammatory responses (1). Currently, the worldwide prevalence of GA ranges from approximately 0.1% to 10%. Partly owing to suboptimal treatment strategies, this disease places a considerable strain on public health systems (2). Current clinical drugs for gout mainly include urate-lowering agents for long-term control and anti-inflammatory agents for acute flares. Urate-lowering therapies target the metabolic basis of gout and reduce recurrence in the long term, but require dose titration and may trigger flares during initiation; by contrast, agents used during acute flares act rapidly to relieve inflammation and pain, but they do not eliminate urate burden or modify the long-term course of the disease. Therefore, a deeper understanding of the underlying pathogenic mechanisms and the development of more reliable experimental models are urgently needed to improve therapeutic strategies.

Ideal models are indispensable for investigating pathological mechanisms, discovering drug targets, and evaluating new therapies prior to clinical trials. There exists widespread scientific agreement that the initiation of GA is triggered by an imbalance in purine metabolite homeostasis, characterized by excessive urate production relative to renal excretion capacity. Thereafter, the deposited MSU crystals activate an innate immune response (e.g., the NLR family pyrin domain-containing 3 (NLRP3) inflammasome) and trigger PCD, such as pyroptosis and NETosis, ultimately leading to a sequence of inflammatory reactions. Therefore, a critical evaluation of existing models based on these core pathogenic mechanisms, coupled with the development of novel models that faithfully recapitulate key clinical features, is crucial for advancing drug discovery and improving clinical management. Although numerous studies and reviews have explored individual aspects of GA, such as uric acid metabolism, inflammasome activation, or specific experimental models, a systematic integration of these dimensions remains insufficient. In particular, the interplay between metabolic dysregulation, innate immune responses, and multiple PCD pathways, as well as their relevance to model construction and translational research, has not been comprehensively summarized. Therefore, this review aims to provide an integrated perspective by linking pathogenic mechanisms with experimental modeling strategies, thereby offering a more coherent framework for understanding GA and facilitating the development of more predictive and clinically relevant models.

## Pathogenesis of GA

### Uric acid and monosodium urate crystals activate the innate immune system

Hyperuricemia forms the metabolic basis for the onset of GA but is not synonymous with GA. Within the physiological ranges, uric acid functions as a vital antioxidant in plasma. It efficiently eliminates reactive oxygen species (ROS) and maintains the intracellular redox equilibrium. However, when serum uric acid concentrations exceed its antioxidant capacity, it starts to promote oxidative stress. This effect is mediated by the activation of NADPH oxidase and xanthine oxidase and by the induction of mitochondrial dysfunction, which collectively lead to excessive ROS generation. Elevated intracellular ROS levels promote the dissociation of thioredoxin-interacting protein (TXNIP) from its binding partner, thioredoxin (TRX). This, in turn, facilitates the interaction of TXNIP with NLRP3, thereby triggering activation of the NLRP3 inflammasome. As an endogenous damage-associated molecular pattern (DAMP), this process drives the persistence and progression of systemic inflammation. When serum uric acid becomes supersaturated, crystallization of MSU initiates, predominantly within synovial joints and adjacent periarticular structures, including tendons and bursae. Subsequently, crystal deposition elicits a direct activation of innate immune pathways, prompting the release of pro-inflammatory effectors, notably interleukin-1β (IL-1β). This process ultimately results in acute arthritis flares (3).

Acting as a DAMP, MSU crystals are detected by innate immune cells through pattern recognition receptors (PRRs), including members of the Toll-like receptor (TLR) family, and thereby directly initiating an immune response (4). Via the adaptor molecule myeloid differentiation primary response gene 88 (MyD88), the TLR signaling pathway triggers simultaneous activation of the nuclear factor kappa B (NF-κB) signaling cascade and the mitogen-activated protein kinase (MAPK) family pathways. This critical activation significantly enhances the transcriptional induction of key NLRP3 inflammasome constituents, including pro-interleukin-1β (pro-IL-1β), tumor necrosis factor-alpha (TNF-α) as well as other pro-inflammatory factors, laying the molecular foundation for the ensuing inflammatory burst.

Phagocytosed MSU crystals induce intracellular stress signals, including lysosomal rupture, potassium ion (K^+^) efflux, and mitochondrial ROS generation. These signals collectively activate NLRP3 inflammasome assembly, leading to caspase-1 activation. Upon activation, caspase-1 enzymatically processes the precursor pro-interleukin-1β (pro-IL-1β) into its biologically active form, mature IL-1β, which is then secreted into the extracellular milieu, triggering a robust inflammatory response (5). IL-1β and TNF-α, as secreted pro-inflammatory mediators, induce endothelial cell activation through paracrine signaling pathways, thereby promoting the secretion of chemokines, such as interleukin-8 (IL-8), and facilitating the massive recruitment of neutrophils into the joint cavity. Although activated neutrophils attempt to phagocytose the crystals, they release additional inflammatory mediators, such as ROS and proteases, which exacerbate tissue damage (6, 7).

**Figure 1:**
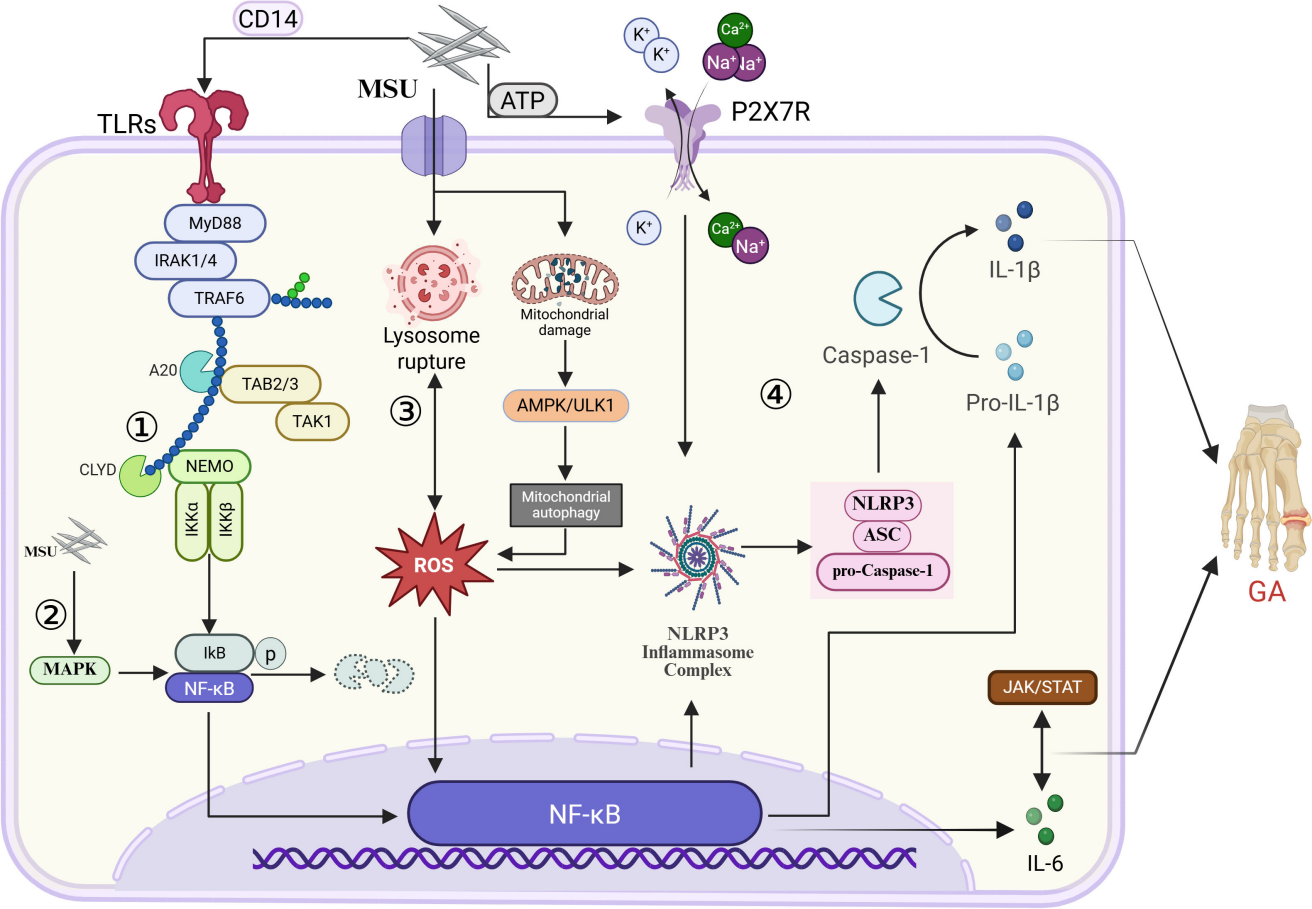
Pathway of innate immune system activation mechanisms. ① MSU indirectly or directly activates TLR signaling pathways via CD14, with downstream signal transduction mediated by MYD88, leading to NF-κB activation and subsequent pro-IL-1β synthesis. ② MSU activates MAPKs, promoting ubiquitin-mediated degradation of inhibitor of κB (IκB). This results in NF-κB liberation and nuclear translocation, thereby mediating inflammatory responses. ③ MSU crystals induce lysosomal rupture, while ROS accumulation causes lysosomal membrane permeabilization (LMP), both of which contribute to inflammatory signaling. ④ MSU elevates extracellular ATP levels, activating P2X purinoceptor 7 (P2X7R) and triggering significant potassium (K^+^) efflux. This process accelerates NLRP3 inflammasome assembly, leading to increased caspase-1 production and subsequent maturation and release of mature IL-1β.

Moreover, MSU triggers the release of pro-inflammatory cytokines, such as interleukin-6 (IL-6), through the NF-κB and Janus kinase/signal transducer and activator of transcription (JAK/STAT) signaling cascades, consequently amplifying the infiltration of inflammatory cells (8). To counteract excessive inflammation, the host initiates negative feedback mechanisms mediated by nuclear factor erythroid 2-related factor 2 (Nrf2)-driven antioxidant pathways, transforming growth factor-β (TGF-β), and natural killer (NK) cells, aiming to limit hyperinflammatory responses (9). However, under chronic hyperuricemia, persistent MSU deposition induces mitochondrial dysfunction and excessive ROS production. Impaired AMP-activated protein kinase (AMPK)/unc-51-like autophagy-activating kinase 1 (ULK1)-mediated mitophagy fails to clear these damaged mitochondria in a timely manner, resulting in sustained NLRP3 inflammasome activation. This process not only exacerbates the release of pro-inflammatory cytokines but, more critically, initiates an inflammatory subtype of PCD termed pyroptosis, characterized by caspase-1-dependent membrane pore formation and pro-inflammatory cytokine release. Together, oxidative stress, NLRP3 inflammasome activation, pyroptosis, and inflammatory mediator release form a self-perpetuating inflammatory loop that drives GA progression (10) (**Figure 1****).**

### Synergistic regulation of inflammation by PCD mechanisms

**Figure 2:**
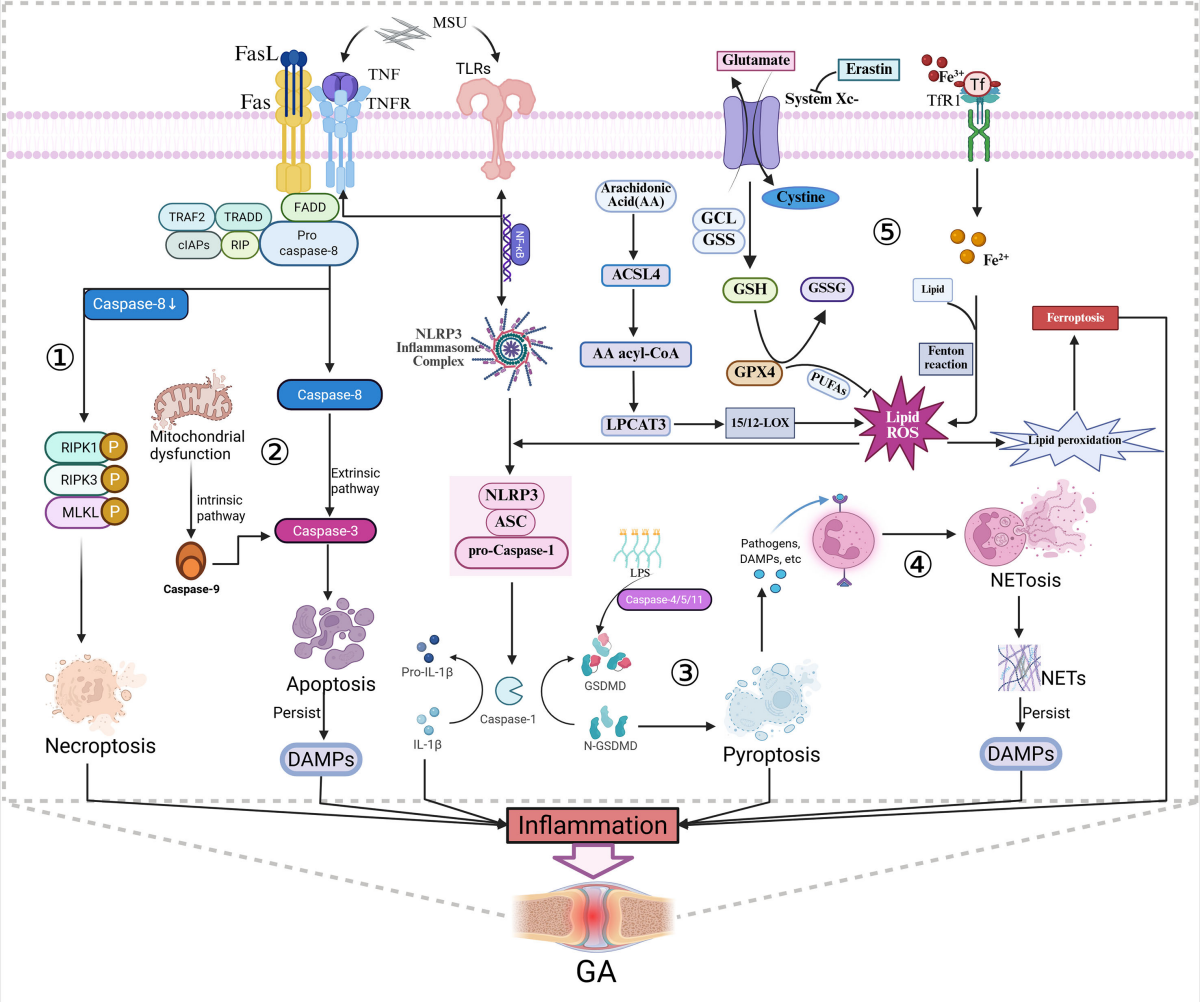
Mechanisms associated with PCD. ①When the activity of Caspase-8 is suppressed, proteins such as FADD will instead activate RIPK1/RIPK3 to form the necrosome. The activated MLKL oligomerizes and disrupts the cell membrane, resulting in necroptosis and the release of pro-inflammatory cytokines. ② Apoptosis is triggered externally by death ligands binding to FADD protein, which then activates Caspase-8. Activated Caspase-8 specifically cleaves Caspase-3 to execute apoptosis. Mitochondrial dysfunction triggers the intrinsic pathway, activating Caspase-9 which in turn activates Caspase-3.③ The NLRP3 inflammasome activates Caspase-1, while LPS directly activates Caspase-4/5/11. These two pathways jointly cleave the GSDMD protein, generating the N-GSDMD fragment. This fragment perforates the cell membrane, resulting in pyroptosis. ④ The reticular structure of NETs (neutrophil extracellular traps) encapsulates MSU crystals. Meanwhile, during the NETosis process, a large amount of histones and other DAMPs are released, jointly mediating the inflammatory response in GA. ⑤ Under the action of inducers such as Erastin, the System Xc - Glutathione (GSH) - GPX4 axis is inhibited, leading to lipid peroxidation of polyunsaturated fatty acid phospholipids mediated by Acyl-CoA synthetase 4(ACSL4)/Lysophosphatidylcholine acyltransferase 3(LPCAT3), ultimately triggering iron - dependent cell death.

PCD denotes an actively regulated process of cellular destruction that is precisely regulated by genetic factors, and it plays a pivotal role in maintaining homeostasis in organisms. In the pathogenesis of GA, multiple PCD modalities collectively form a complex immune regulatory network. Pyroptosis, a pivotal component of innate immune responses, directly converts MSU crystal recognition into robust inflammatory cascades. NETosis, a unique form of neutrophil death, not only traps pathogens to limit infection but also exacerbates tissue injury resulting from the release of neutrophil extracellular traps (NETs). Apoptosis, as a non-inflammatory silent death modality, primarily orchestrates the timely resolution of inflammatory responses and restoration of tissue homeostasis. When apoptosis is inhibited, death receptor signaling shifts toward necroptosis, further amplifying inflammatory responses. Recently, ferroptosis, a type of cell death dependent on iron and driven by lipid peroxidation, has been drawing growing interest due to its possible involvement in the progression of GA and ongoing joint damage. These distinct PCD mechanisms interact synergistically, collectively determining the inflammatory trajectory and clinical outcomes of GA (**Figure 2**).

## Pyroptosis

Pyroptosis represents a form of programmed inflammatory cell death orchestrated by the gasdermin (GSDM) protein family, with gasdermin D (GSDMD) playing a predominant role. The central mechanism involves caspases-mediated cleavage of GSDMD, resulting in the release of its N-terminal fragment (GSDMD-N). This fragment then oligomerizes to form pores in the plasma membrane. This disruption upsets the osmotic balance, which in turn causes the cell to swell, rupture, and release large quantities of pro-inflammatory cytokines.

In GA, the key upstream initiating signal for pyroptosis is the NLRP3 inflammasome-mediated activation of caspase-1. Activated caspase-1 performs dual functions: on one hand, it cleaves GSDMD, generating the pore-forming N-terminal fragment (GSDMD-N), which oligomerizes within the plasma membrane, facilitating pore formation (10-20 nm in diameter) and directly inducing pyroptotic cell death (11). On the other hand, it converts the precursors of pro-inflammatory cytokines, such as IL-1β and IL-18, into their mature, active forms. Notably, the GSDMD pores serve as channels for the release of these macromolecules, thereby amplifying robust inflammatory signals and releasing them into the extracellular space. Overall, current evidence for pyroptosis in GA is supported mainly by cellular and animal studies, while direct clinical validation remains limited.

Pyroptosis can occur via both canonical and non-canonical pathways. The canonical pathway is primarily triggered by MSU crystals through NLRP3 inflammasome activation and caspase-1 signaling, whereas the non-canonical pathway is mediated by caspase-4/5 in humans (caspase-11 in mice) in response to intracellular LPS. Although MSU does not directly activate the non-canonical pathway, both pathways converge on GSDMD cleavage, leading to membrane pore formation. Moreover, non-canonical activation can further enhance NLRP3 inflammasome signaling, thereby amplifying IL-1β release and inflammatory responses. Consequently, non-canonical pyroptosis may act as an auxiliary amplifier in the inflammatory microenvironment of GA. Damage-associated molecular patterns (DAMPs) released from pyroptotic cells further promote neutrophil activation and NETosis, contributing to sustained inflammation (10).

## The dual roles of NETosis

NETosis represents a specialized form of neutrophil cell death triggered by cellular activation, in which neutrophils release web-like structures composed of DNA, histones, and antimicrobial proteins as a defense mechanism against infection. In GA, NETosis exerts dual regulatory effects on inflammation.

On the one hand, NETs physically entrap MSU crystals via their meshwork, limiting crystal dissemination and suppressing excessive inflammation by degrading pro-inflammatory mediators, thereby contributing to the containment of inflammation. On the other hand, NETosis releases substantial amounts of inflammatory mediators, including histones and myeloperoxidase (MPO). Notably, histones and other NET components function as DAMPs, further amplifying NLRP3 inflammasome activation and pyroptosis in macrophages, thereby perpetuating a vicious inflammatory cycle (12).

Critically, direct interplay exists between pyroptosis and NETosis: membrane pores formed by GSDMD activate neutrophil elastase (NE), which in turn cleaves GSDMD to promote NETosis (13). Concurrently, components within NETs, such as matrix metalloproteinase-9 (MMP-9), directly degrade cartilage matrix, leading to irreversible joint damage.

## Apoptosis

In contrast to the pro-inflammatory properties of pyroptosis and NETosis, PCD in the form of apoptosis acts in an immunologically silent manner and plays a critical regulatory role during the resolution phase of GA inflammation. The process is triggered by either the extrinsic (death receptor) or intrinsic (mitochondrial) pathway, resulting in caspase-3 activation, cellular shrinkage, membrane blebbing, and apoptotic body formation. Phagocytes efficiently clear these bodies without triggering inflammatory responses (14).

In GA, timely apoptosis of macrophages helps terminate the sustained release of pro-inflammatory signals. Active clearance of apoptotic cells by phagocytes inhibits the production of pro-inflammatory cytokines, such as IL-1β, IL-8, and TNF-α, and increases the secretion of anti-inflammatory mediators such as transforming growth factor-β1 (TGF-β1), prostaglandin E2 (PGE2), and platelet-activating factor (PAF), ultimately leading to the resolution of inflammation (15). However, the intense inflammatory microenvironment induced by MSU crystals, pyroptosis, and NETosis disrupts this protective mechanism through two pathways. First, it impairs phagocytic function, leading to secondary necrosis of apoptotic cells, which subsequently converts into pro-inflammatory signals. Second, it may also impede the functioning of caspase-8, a crucial activator of apoptosis, forcing cells to shift toward the more pro-inflammatory necroptotic cell death pathway. These mechanisms collectively underpin the pathogenesis of GA (16).

## Necroptosis

Necroptosis is a regulated form of necrotic cell death, which is mediated by receptor-interacting protein kinases 1 and 3 (RIPK1/RIPK3), which activate their key effector, mixed lineage kinase domain-like pseudokinase (MLKL). When caspase-8 activity is suppressed in the GA microenvironment, death receptor (e.g., tumor necrosis factor receptor 1 (TNFR1)) signaling shifts from apoptosis to the necroptotic pathway. Once activated, MLKL molecules cluster together (oligomerize) and render the plasma membrane permeable. This results in the discharge of cellular contents and a consequent intensification of inflammatory reactions. In GA, necroptosis may synergize with pyroptosis to form the cellular basis of acute-phase hyperinflammation, providing a mechanistic explanation for sustained inflammation. Thus, necroptosis likely represents a critical mechanism underlying the onset and progression of GA.

## Ferroptosis

Ferroptosis is a type of PCD that is triggered by the accumulation of lipid peroxides and is dependent on iron, with its core mechanism involving the inactivation of the glutathione peroxidase 4 (GPX4)-centered antioxidant defense system. During the intercritical phase of GA, persistent oxidative stress leads to significant depletion of glutathione. This may directly inhibit GPX4 activity, thereby impairing cellular capacity to eliminate lipid peroxides and ultimately inducing ferroptosis (17). Ferroptotic cells release multiple pro-inflammatory signals, including lipid peroxidation end-products, which sustain activation of the NLRP3 inflammasome. Ferroptosis has the potential to work in concert with alternative cell death pathways, for instance pyroptosis, to perpetuate local inflammatory states, drive synovial fibrosis, and promote bone erosion, playing a pivotal role in GA progression (17, 18) (**Figure 2**).

Taken together, GA is a multifactorial disease involving metabolic abnormalities, crystal deposition, inflammatory amplification, and the interplay among multiple forms of programmed cell death (PCD), which underscores the need to establish appropriate animal models capable of recapitulating these interconnected pathological events.

## Experimental animal models of GA

Accurate animal models that can replicate the key pathological features of GA are essential for thorough research into the mechanisms of GA and the evaluation of therapeutic strategies.

An ideal model should meet the following requirements: Firstly, it should be able to simulate the metabolic disorder by using drugs to inhibit uric acid excretion. Secondly, it should facilitate the formation of MSU crystals within joints, which serves as the physical basis for immune activation. Thirdly, it should replicate key events, including the triggering of the NLRP3 inflammasome, the secretion of IL-1β, and the infiltration of neutrophils, and present persistent inflammation and tissue destruction resulting from the dysregulation of the PCD network.

Based on the aforementioned pathological characteristics, this review will systematically assess the advantages and disadvantages of the existing methods for preparing GA animal models and evaluate their capabilities and values in simulating specific pathological processes.

## Exogenous MSU crystal-induced models: mimicking the acute inflammatory phase

**Figure 3:**
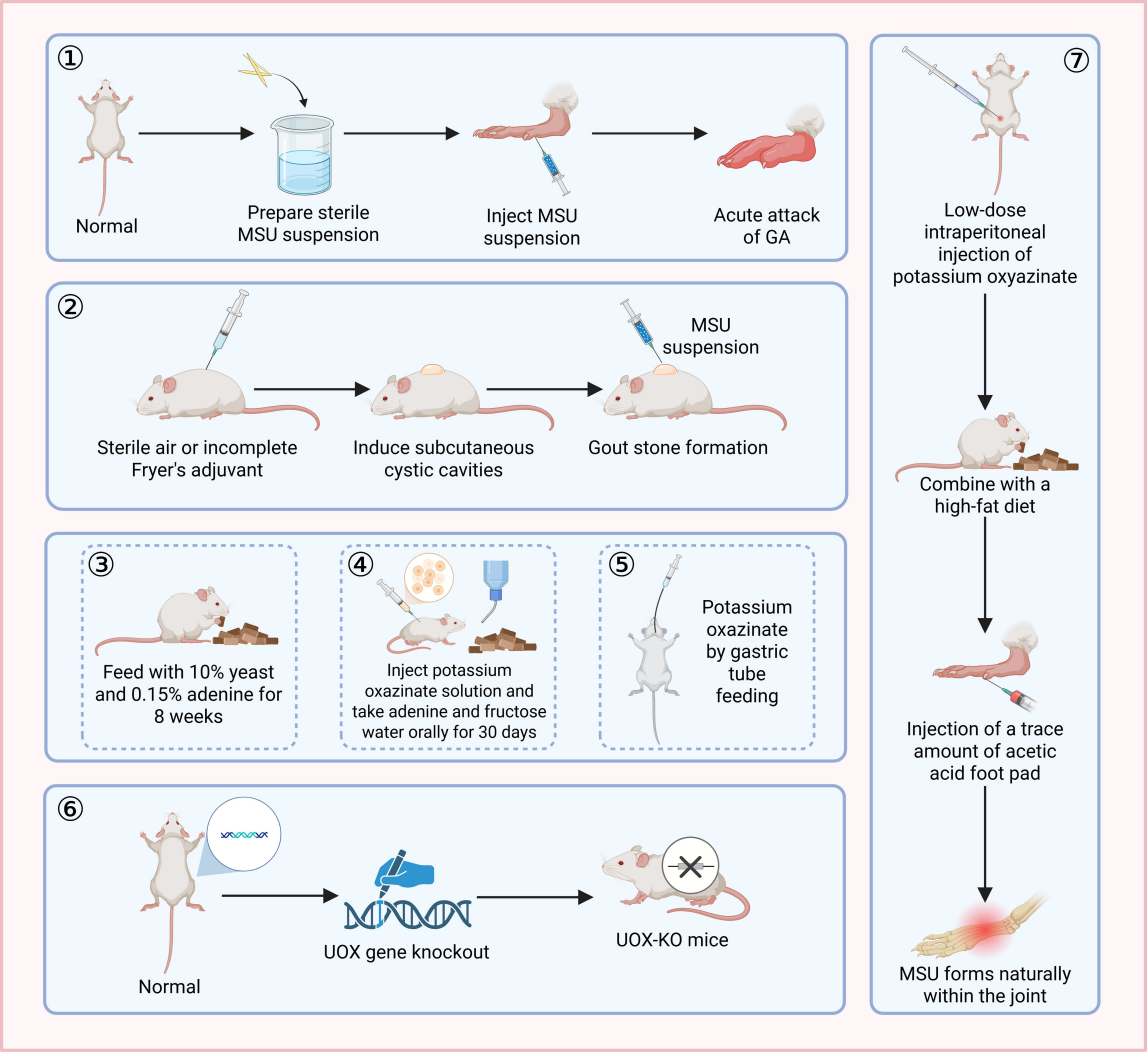
Flowchart of the modeling process for GA. Figure caption: ① Footpad injection model; ②Air pouch injection model; ③④⑤ Hyperuricemia-inducing models; ⑥ UOX-KO mouse model; ⑦ Composite model with spontaneous formation of MSU crystals.

Exogenous MSU crystal-induced models bypass the metabolic process by directly injecting MSU crystals. Focusing on the acute innate immune response triggered by MSU crystals, these models serve as classic tools for investigating the mechanisms underlying inflammatory flare-ups (19, 20). However, such models are mainly characterized by lymphocyte infiltration, which differs from the neutrophil-dominated feature in the acute phase of human GA. Moreover, there is a potential risk of traumatic inflammation (infection) caused by mechanical injury (21). The footpad injection model involves administering MSU crystals into either the subcutaneous or muscular layer of the hind footpad of mice. This model can elicit typical neutrophil infiltration and edema. It is easy to perform and suitable for high-throughput screening of anti-inflammatory drugs (22). The air pouch injection model requires the subcutaneous injection of sterile air (1-2 mL) or Freund’s incomplete adjuvant into the back of rats or mice 3-7 days prior to modeling to induce the formation of a pouch. Once the pouch is mature, an MSU suspension is injected into it. This model is applicable to the study of tophus formation and associated synovitis. However, due to the lack of bone erosion pathology, it is not suitable for research on bone damage (23, 24). The minimally invasive embedding technique has revolutionized the method of crystal delivery. Xu et al. (25) directly embedded MSU crystals into the rat articular cavity instead of injecting a suspension, achieving sustained inflammation lasting over 96 hours and high local expression of IL-1β/TNF-α. This significantly addresses the issue of rapid crystal clearance in traditional models (**Figure 3**) (**Table 1****).**

**Table 1:** *In vivo* animal experimental models.

Model category	Representative animals	Induction methods	References
Exogenous MSU Crystal-Induced Models	Male white albino rats	Prepare a 20 mg/mL MSU suspension and administer a single 0.5 mL injection into the right hindlimb knee joint.	(19)
	Rat	Coderre’s method: Administer a 1.25 mg bolus of MSU microcrystals via intra-articular injection into the ankle cavity.	(20)
	C57BL/6 mice	Inject MSU (50 mg/mL, 40 μL) into the footpad.	(22)
	Rats/mice	After pre-inducing an air pouch by subcutaneously injecting 1-2 mL of sterile air into the back 3-7 days prior, inject MSU into the pouch.	(23)
	C57BL/6 mice	MSU microcrystals (0.5 mg in 20 μL sterile phosphate-buffered saline (PBS)) were injected subcutaneously into the plantar surface of the hind footpad.	(24)
Hyperuricemia and Endogenous Crystal Deposition Models	ApoE-deficient mice	Administer PO (100 mg/kg) via oral gavage.	(26)
	Sprague-Dawley (SD) rats	Feed the animals a regular diet supplemented with 10% yeast and 0.15% adenine for 8 weeks.	(27)
	SD rats	Subcutaneously inject a PO solution at a dose of 0.5 g/kg/day, orally administer adenine at a dose of 0.1 g/kg/day, and provide free access to a 20% fructose-containing water solution for 30 days.	(28)
Composite Models	C57BL/6 mice	The animals were fed a high-fat diet (20% sucrose, 15% lard, 0.8% cholesterol, 0.2% sodium cholate, plus casein, calcium phosphate, and minerals) by oral gavage (0.1 mL/g body weight) from postnatal day 14 to 49. Starting on day 7, MSU crystals (1 mg in 40 μL sterile PBS) were injected subcutaneously into the right hind footpad every 10 days.	(30)
	Male SD Rats	The rats were maintained on a high-cholesterol diet (30 g/kg cholesterol, 100 g/kg egg yolk powder) and alternately gavaged every other day with white wine, lard, or honey water (1.5 mL/100 g body weight). On day 18, an MSU suspension (150 μL) was injected into the right ankle joint cavity.	(31)
	Male Kunming mice	The mice were administered daily intraperitoneal injections of low-dose PO (150 mg/kg) and fed a high-fat diet (60% kcal from fat). Concurrently, bilateral hind paws were injected twice weekly with 20 μL of 0.1% acetic acid for 4 months to induce the model.	(33)

## Hyperuricemia and endogenous crystal deposition models: reflecting metabolic initiation and disease progression

These models are designed to mimic the metabolic initiating process of GA by intervening in uric acid metabolism to induce endogenous hyperuricemia. By administering uricase inhibitors (such as potassium oxonate (PO)) or providing high-purine/high-yeast diets, the excretion of uric acid can be effectively inhibited or its production can be promoted, thereby establishing hyperuricemia. However, these models lack typical inflammatory manifestations (26, 27). Using a combination of compounds (such as PO, fructose, and adenine) can result in a more stable increase in serum uric acid levels and trigger renal problems that lead to functional impairment, which is more characteristic of hyperuricemia in the context of human metabolic syndrome (28). Although uricase knockout (UOX-KO) mice simulate the uric acid metabolic defects in humans, this model is often associated with high mortality rates and severe renal damage. Moreover, joint inflammation caused by MSU crystal accumulation within the joint space is rarely observed, possibly because severe renal damage leads to premature death of the models before arthritis sets in, thereby limiting its widespread use (29) (**Figure 3**) (**Table 1**).

## Composite models: integrating metabolic abnormalities and acute inflammation

Composite models simultaneously simulate metabolic disorders and acute inflammation by applying exogenous MSU crystal stimulation in the context of hyperuricemia. Human GA is typically not an acute inflammatory condition triggered by a single factor; rather, it is a disease closely associated with long-term hyperuricemia, unfavorable living factors (for instance, consuming high-fat foods and drinking alcohol), and metabolic syndrome. Composite models more closely resemble the pathogenesis of clinical GA. These models are commonly constructed by feeding animals a high-fat diet combined with MSU crystal injection (30). Li et al. (31), further incorporated interventions such as white wine, lard, and honey water in addition to the high-fat diet, successfully establishing a composite model with both metabolic abnormalities and severe inflammation. Lin et al. (32), observed more severe systemic inflammation and gut microbiota dysbiosis in this model. However, the modeling process is time-consuming, typically requiring more than 6 weeks. Precise control of uric acid levels and injection timing is necessary, and the operational complexity limits its widespread application. Recent breakthroughs have been made in novel composite models. Wang et al. (33) achieved the natural formation of MSU crystals in animal joints for the first time by administering long-term low-dose intraperitoneal injections of PO, combined with a high-fat diet and trace amounts of acetic acid injected into the footpad. They also observed bone erosion and synovial hyperplasia, providing an ideal platform for studying the natural course of GA and anti-ga drugs (**Figure 3****) (****Table 1**).

## Application and comparison of different animal species

Due to differences in metabolic characteristics, different species have distinct focuses in the study of GA models.

## Avian species

Chickens (34), quails (35), geese, and birds (such as red-tailed hawks) lack functional uricase (UOX), resulting in a high degree of similarity in uric acid metabolism to that of humans. Hyperuricemia and articular urate deposition can be naturally induced in these species through high-calcium or high-protein diets, making them ideal models for studying long-term metabolic disorders. Moreover, chickens, ducks, and geese can spontaneously develop GA upon infection with avian astrovirus (36). However, there are several limitations associated with these models, including high breeding costs, significant fluctuations in uric acid levels, and substantial biological differences from humans. Additionally, once GA develops, it often progresses rapidly to visceral GA, leading to a high mortality rate, which significantly restricts the application of these models (**Table 1**).

## Rodents

Rodents primarily consist of rats, mice, and rabbits. Due to their low cost and the feasibility of standardized operations, rats and mice are extensively employed in exogenous MSU crystal-induced models and hyperuricemia-inducing models. However, one limitation is that rodents possess UOX in their bodies, necessitating the inhibition of UOX activity to mimic human metabolic defects. Rabbits (23) also have UOX in their bodies and thus require exogenous modeling. The classic approach involves inducing inflammation by injecting MSU crystals into the knee joint. Compared to rats and mice, rabbits exhibit a lower degree of pathological simulation and involve a more complex operational process (37) (**Table 1**).

## Other animals

Non-human primates, such as rhesus monkeys, share homologous uric acid metabolism to that of humans. However, their application is limited due to exorbitant costs and difficulties in achieving standardization of strains. Other species, including zebrafish and tree shrews, have been utilized in preliminary studies of hyperuricemia owing to their unique advantages, such as rapid reproduction and genomic similarity to humans. For instance, hyperuricemia can be induced in juvenile zebrafish by using PO or sodium xanthine, but these models still require further validation as GA models (38, 39). The pig model has confirmed the existence of an intestinal uric acid excretion pathway (40). The method of injecting MSU crystals into canine joints pioneered the research on GA models, but it has gradually been replaced by rodent models due to cost and ethical constraints (41). Each of these models has its own characteristics and provides diverse tools for the study of GA mechanisms and drugs, yet all of them have limitations (**Table 1**).

## *In vitro* cellular models of GA

Animal models offer a comprehensive view for *in vivo* research on GA, whereas *in vitro* cellular models enable the elucidation of the mechanisms underlying innate immune activation and PCD at the molecular and cellular levels by precisely controlling the cellular microenvironment. These models serve as a crucial platform for high-throughput drug screening. Their primary advantage lies in directly pinpointing a cause-and-effect link between MSU and particular cell types. However, the simplified nature of these models and their differences from the complex *in vivo* pathological conditions should be carefully considered. Based on the complexity of the system, *in vitro* cellular models can be classified into single-cell culture systems and co-culture systems.

## Single-cell culture systems

This system is designed to clarify the specific action pathways of particular cell types in GA through their isolation.

**Figure 4:**
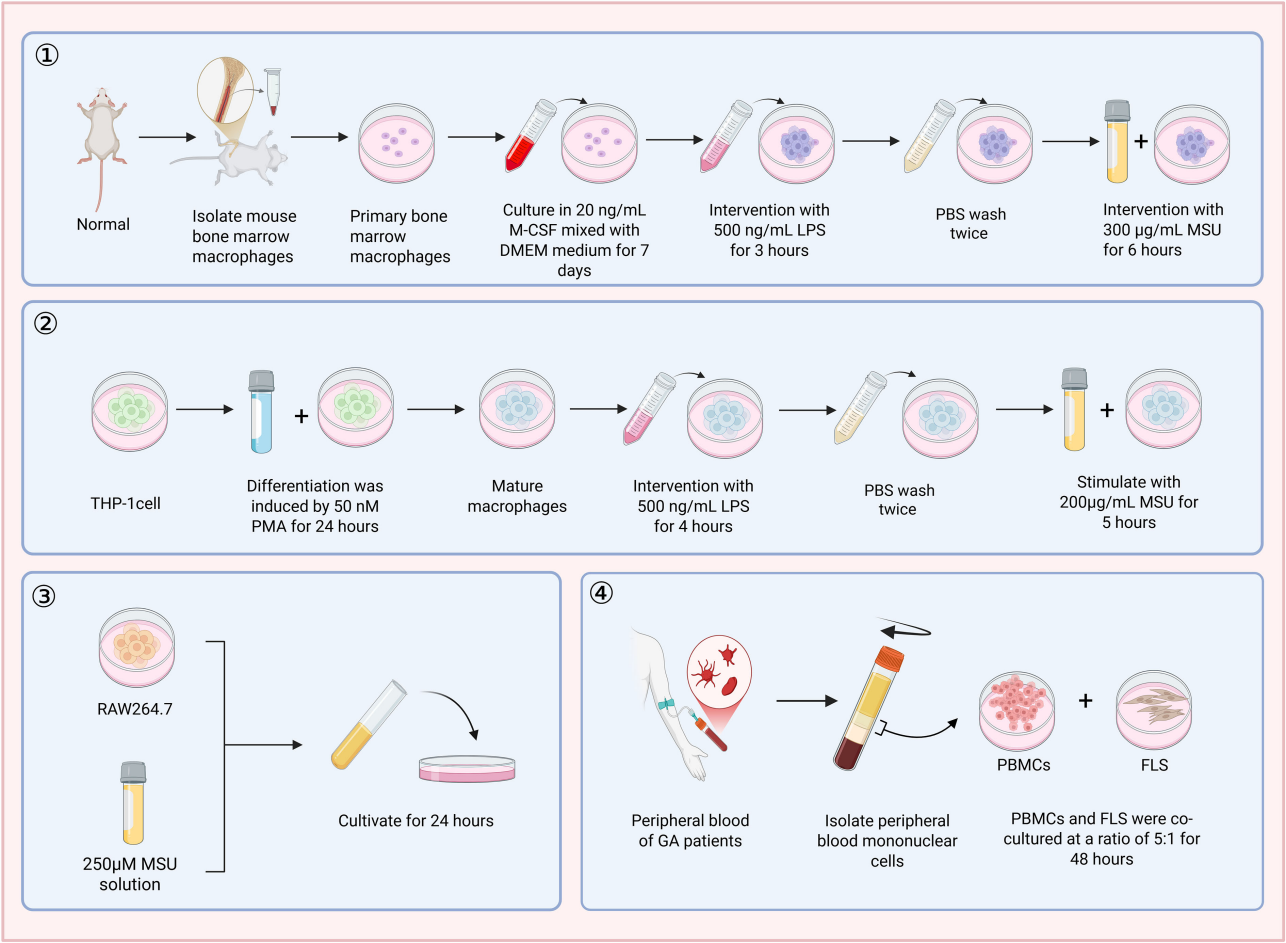
Modeling process of *in vitro* cell models. Single - cell culture systems: ① Mouse primary bone marrow - derived macrophage model; ② THP - 1 cell model; ③ RAW264.7 cell model.Co - culture system. Co - culture systems: Mononuclear cells derived from the peripheral blood (PBMCs), which are separated out from the blood circulating around the body of individuals with GA and FLS.

The monocyte-macrophage model, which serves as the core executor of NLRP3 inflammasome activation, IL-1β release, and pyroptosis, is the focus of research. Among primary cells, bone-marrow-derived macrophages (BMDMs) can highly mimic the *in vivo* pro-inflammatory state and polarization; however, their preparation process is complex (42–44). Peritoneal macrophages (MPMs) are mainly obtained from mouse peritoneal lavage fluid and are rich in tissue-resident macrophages. They respond rapidly and are suitable for short-term studies, but their low yield limits their large-scale application (45). Jiang et al. (46) used a model of peritoneal macrophages induced by LPS pre-treatment combined with MSU, which effectively simulated acute inflammation and was used for polarization research. Among immortalized cell lines, mouse-derived RAW264.7 reportedly lacks or express very low levels of apoptosis-associated speck-like protein containing a caspase recruitment domain (ASC). Their ability to assemble the canonical NLRP3 inflammasome is limited, which makes their application in canonical pyroptosis-related studies controversial. Therefore, RAW264.7 cells are more suitable for preliminary screening or general inflammatory-response studies, and key findings should ideally be validated in primary macrophages or other more appropriate cell systems. They are commonly stimulated with MSU, LPS, or a combination of both (47, 48). In addition, the murine macrophage-like J774 (J774A.1) cell line has been reported to be a useful *in vitro* model for GA research. Owing to its stable growth characteristics and responsiveness to inflammatory stimulation, it can serve as a complementary tool for investigating MSU-induced macrophage activation and inflammatory signaling (49). The Tsuchiya human monocytic leukemia line (THP-1) has the advantage of a human background and is suitable for research on human-specific drug mechanisms. However, its ability of secrete factors such as IL-1β is relatively weak, and conclusions need to be verified with primary cells. It is commonly modeled using a combination of PMA and MSU (50, 51) (**Figure 4****) (****Table 2**).

**Table 2:** Overview of *in vitro* cell models used in the study.

*In vitro* model categories	Cell selection	Induction methods	References
Single-cell Culture Systems	BMDMs	Incubation with M-CSF combined with IL-1β.	(43)
	BMDMs	Cells were cultured in DMEM medium mixed with 20 ng/mL M-CSF. On day 7, cell samples were treated with 500 ng/mL LPS for 3 hours, followed by two washes with PBS, and then the cells were treated with 300 μg/mL MSU for 6 hours.	(44)
	MPMs	Cells were pre-stimulated with LPS and then induced with MSU.	(46)
	RAW264.7	Stimulation with LPS (100 ng/mL) for 24 hours.	(47)
	RAW264.7	Stimulation with 250 μM MSU for 24 hours.	(48)
	J774	LPS priming (100 ng/mL for 1 h), followed by MSU crystal stimulation (200 μg/mL for 6 h)	(49)
	THP-1	Treatment with 50 nM PMA for 24 hours to induce differentiation into macrophages. Then cells were pre-treated with 500 ng/mL lipopolysaccharide (LPS) for 4 hours and stimulated with 200 μg/mL MSU for 5 hours.	(50)
	THP-1	Stimulation with 0.5 μM PMA for 3 hours, followed by cell washing and stimulation with 250 μg/mL MSU for 24 hours.	(51)
	Neutrophils	Cells were induced by MSU and Phorbol Myristate Acetate (PMA).	(52)
	Neutrophils	Cells were induced by stimulation with 100-200 μg/mL MSU for 2-4 hours.	(16, 53)
	Human fibroblast-like synoviocytes (MH7A cells)	Intervention with 100 ng/mL LPS for 90 minutes, followed by treatment with 150 μg/mL MSU for 24 hours.	(54)
	Mouse chondrocytes	Stimulation with MSU solution (100 μg/mL).	(68)
	C28/I2 cells	Incubation with MSU (200 μg/mL) for 24 hours.	(55)
	HUVECs	Stimulation with 100 μg/mL MSU for 24 hours.	(57)
	HK-2 cells	Intervention with MSU.	(59)
	HK-2 cells	Incubation with 2.5 mmol/L adenosine solution for 24 hours, followed by intervention with 0.005 IU/mg xanthine solution for 12 hours.	(60)
Co-culture Systems	Chondrocytes-peritoneal macrophages	Stimulation with LPS (1 μg/mL) for 4 h, followed by stimulation with MSU (0.2 mg/mL) for 18 h.	(46)
	Adipose-derived Mesenchymal Stromal/Stem Cell (Ad-MSC)-THP-1	Induction with MSU.	(61)
	Neutrophils-synovial fibroblasts	Collect the mouse bone marrow washings, filter them, and then separate neutrophils by gradient centrifugation. After culturing neutrophils with 0.2 mg/mL MSU for 24 hours to obtain the supernatant, the neutrophils were co-cultured with synovial fibroblasts.	(62)
	PBMCs-FLS from GA patients	Co-culturing at a ratio of 5:1 for 48 h.	(63)

Neutrophil models are isolated from human or mouse peripheral blood. Stimulation with MSU can rapidly induce mitochondrial autophagy, NET formation, and the release of pro-inflammatory factors, making them an ideal tool for studying acute-phase responses and NETosis. However, their short survival time limits their use to short-term experiments (52, 53).

Synovial cell models are mainly derived from primary cells of human or mouse synovial tissues and fibroblast-like synoviocyte (FLS) cell lines. They can directly reflect the damage of MSU to joint tissues and are suitable for drug intervention research. However, primary cells are difficult to obtain, and cell lines may lose *in vivo* characteristics. Li et al. (54) successfully established an *in vitro* model using MH7A cells.

Chondrocyte models are derived from primary chondrocytes of animals such as rabbits and mice or the human-derived C28/12 chondrocyte cell line. They are used to study urate-induced cartilage degradation and autophagy abnormalities. Primary cells have high physiological relevance, while cell lines are convenient for standardized screening. MSU crystals or a combination of IL-1β/TNF-α are commonly used to induce cartilage degradation and autophagy abnormalities (55, 56).

Human umbilical vein endothelial cells (HUVECs) are derived from human umbilical vein endothelial tissues and are used to evaluate the inflammatory damage of MSU to the endothelium and the protective effect of drugs. Usually, a model is established by stimulating HUVECs with MSU for 24 hours. Pen et al. (57) established a model by treating HUVECs with 100 μg/mL MSU for 6 hours. This model can directly reflect the inflammatory damage of urate to the endothelium and is suitable for evaluating the endothelial-protective effect of drugs. It is mainly used in the research on the mechanisms of GA-related vascular complications. However, as primary cells, they have limited passage capacity and cannot fully reproduce the *in vivo* vascular microenvironment.

Mesenchymal stem cells from the human umbilical cord (HUC-MSCs) have shown significant anti-inflammatory and immune-regulating properties in preclinical models of rheumatoid arthritis (RA). Arthritic pathology is improved by these cells through a variety of mechanisms, including macrophage phenotype alteration (towards anti-inflammatory M2 polarization), inhibition of pro-inflammatory cytokine release (for example, TNF-α, IL-6), and promotion of regulatory T cell (Treg) development. Notably, extracellular vesicles secreted by HUC-MSCs show comparable therapeutic efficacy in GA by regulating macrophage activation states and inhibiting the production of inflammatory mediators (58).

The HK-2 cell model, originating from a human renal proximal tubular epithelial cell line, serves as a valuable *in vitro* system for studying renal urate handling. Meng et al. (59) developed a gout-related inflammation model by stimulating HK-2 cells with monosodium urate (MSU) crystals, while Hou et al. (60) established a hyperuricemia model through adenosine/xanthine treatment. This cellular platform enables specific investigation of proximal tubular epithelial cell functions in uric acid metabolism, offering the advantages of rapid experimental turnover and high-throughput capacity. Consequently, it is widely utilized for preliminary mechanistic studies of hyperuricemic nephropathy and efficacy evaluation of urate-lowering agents.

## Co-culture systems

To overcome the limitations of single-cell models, complex systems such as co-culture have been developed to simulate intercellular communication.

The co-culture of macrophages and chondrocytes directly validated the IL-1β-mediated cartilage catabolic axis. Jiang et al. (46) co-cultured peritoneal macrophages with rat chondrocytes that were co-stimulated with LPS and MSU for subsequent pharmacological research.

The macrophage-mesenchymal stem cell (MSC) co-culture model can be used for both pathogenesis research and the validation of therapeutic strategies (58). Rocha et al. (61) established a GA model by co-culturing adipose-derived MSCs (Ad-MSCs) stimulated with MSU with macrophages derived from THP-1 cells.

The neutrophil-centered model generates NETs by stimulating neutrophils with MSU, which then activate synovial fibroblasts and other cells to study the role of NETosis in amplifying and maintaining inflammation. Tian et al. (62) stimulated neutrophils with MSU to form NETs and subsequently used them to induce an inflammatory response in synovial fibroblasts.

Complex models based on clinical samples directly utilize primary cells from GA patients, maximally preserving the individual characteristics of the disease. In the patient immune cell-synovial cell co-culture model, monocytes are extracted from the peripheral blood of acute GA patients and co-cultured with synovial cells (63). Schorn et al. (64) directly extracted polymorphonuclear neutrophils (PMNs) from the peripheral blood of GA patients and added MSU crystals for modeling and research. This model can better reflect the patient’s own immune characteristics.

The human synovial fluid primary cell model directly utilizes the cell mixture in the synovial fluid of acute GA patients, which is rich in neutrophils and macrophages. It is a natural co-culture system that most closely resembles the *in vivo* inflammatory microenvironment. By obtaining synovial fluid from acute GA patients through puncture, the cell population obtained after centrifugation (usually rich in 60-90% neutrophils and monocytes/macrophages) itself represents a natural co-culture system that may closely simulate the inflammatory response in the joint cavity (65).

Organoid models, as emerging *in vitro* research platforms, simulate the structure and function of organs *in vivo* through three-dimensional culture techniques and show unique value in GA research. Their core advantage lies in integrating multiple cell types and reproducing intercellular interactions, providing a promising new platform for studying crystal deposition and inflammatory responses in an environment closer to *in vivo* condition. Chen et al. (66) verified, using osteoarthritis synovial organoids, that MSU crystals are more likely to deposit in osteoarthritis synovium compared with normal synovium. Hou et al. (67) established an *in vitro* liver organoid model to simulate cell growth patterns and screen anti-hyperuricemia compounds (**Figure 4****) (****Table 2**).

## Conclusions and future perspectives

GA is a multifactorial disease characterized by the interplay of metabolic dysregulation, MSU crystal deposition, innate immune activation, and multiple forms of PCD—including pyroptosis, NETosis, apoptosis, necroptosis, and ferroptosis. These interconnected processes collectively shape the initiation, amplification, and persistence of inflammation. Current experimental models have substantially advanced mechanistic research on GA. Animal models provide an overall *in vivo* perspective, enabling macroscopic reproduction of pathological features including inflammatory cell infiltration, tissue edema, joint damage, and bone erosion, which is an essential step for evaluating the overall efficacy of drugs. However, their application is fundamentally limited by interspecies differences in metabolism and immunity. Most mammalian models express functional UOX, which prevents the spontaneous development of persistent hyperuricemia and requires exogenous interventions. This introduces non-physiological disturbances and limits the faithful recapitulation of natural MSU crystal deposition in humans. Although composite models better recapitulate disease progression, their complexity, long duration, and high cost severely restrict their use as mainstream platforms for high-throughput screening and mechanistic studies. Furthermore, precise reproduction of the aforementioned pathogenic mechanisms during model establishment is challenging, impeding the development of specific targeted drugs.

In contrast, *in vitro* cell models offer core advantages of high throughput, controllability, and focus. They can directly establish causal relationships between MSU crystals and specific target cells at the molecular and cellular levels, serving as key platforms for clarifying inflammatory mechanisms such as NLRP3 inflammasome activation, pyroptosis, and NETosis, as well as for target validation and high-throughput drug screening. However, their notable limitation lies in over-simplification. Single-cell cultures fail to simulate the complex paracrine and contact-dependent communication among immune cells, stromal cells, and chondrocytes in the joint microenvironment. Although new technologies such as co-culture systems, patient-derived primary cells, and organoids attempt to reconstruct cellular interactions, the complete simulation of critical physiological factors *in vivo*—including tissue hierarchical structure, biomechanical environment, nutrient gradients, and neurovascular regulation—remains a core scientific and technical challenge in current *in vitro* research.

In summary, no single model can fully cover all pathological features of GA. A central implication for model development is that no experimental system should be expected to capture all disease dimensions simultaneously. Instead, pathogenic precision and translational usefulness depend on matching the model to the dominant biological process under study. Importantly, most exogenous MSU injection models primarily reproduce acute inflammatory flares rather than the spontaneous and natural course of human gout (69), whereas hyperuricemia models mainly reflect the metabolic basis of disease initiation and do not directly correspond to typical gouty arthritis attacks. In recent years, hyperuricemia model has proven to be of significant value in assessing uric acid excretion and gut-microbiome interactions (70). Composite models are therefore more informative for studying chronic progression and structural damage, while *in vitro* systems, including RAW264.7 cells, THP-1 cells, BMDMs, co-culture systems, and organoid models, are particularly valuable for mechanistic studies and drug screening. Therefore, future studies should adopt complementary and integrated strategies, combining models of different levels based on specific scientific questions to construct a complete evidence chain from molecular mechanisms to overall pathology. Given the inherent limitations of current cellular and animal models in simulating the complex pathology of GA, the combined use of appropriate cellular and animal models based on experimental objectives may be the optimal choice until new models that overcome existing interspecies differences and technical bottlenecks emerge. Organoid models, which integrate multiple cell types, may become a research focus.

An ideal GA animal model should systematically simulate its core pathogenic mechanisms: from disordered uric acid metabolism and MSU crystal deposition to the activation of the innate immune system, ultimately leading to dysregulation of the PCD network represented by pyroptosis. By integrating the aforementioned strategies to construct a multi-level, high-precision experimental model system that closely approximates the pathological essence of human GA, we are able to not only enhance our comprehension of disease mechanisms, but also substantially expedite the preclinical advancement and translational headway of innovative therapeutic approaches.
